# Virtual monoenergetic images from photon-counting spectral computed tomography to assess knee osteoarthritis

**DOI:** 10.1186/s41747-021-00261-x

**Published:** 2022-02-22

**Authors:** Christine Chappard, Juan Abascal, Cécile Olivier, Salim Si-Mohamed, Loic Boussel, Jean Baptiste Piala, Philippe Douek, Francoise Peyrin

**Affiliations:** 1 B3OA, CNRS UMR 7052, U 1271 Inserm, University of Paris, Paris, France; 2 University of Lyon, INSA-Lyon, CNRS, Inserm, CREATIS UMR 5220, U1206, Lyon, France

**Keywords:** Bone cysts, Cartilage, Osteophyte, Osteoarthritis (knee), Tomography (X-ray computed)

## Abstract

**Background:**

Dual-energy computed tomography has shown a great interest for musculoskeletal pathologies. Photon-counting spectral computed tomography (PCSCT) can acquire data in multiple energy bins with the potential to increase contrast, especially for soft tissues. Our objectives were to assess the value of PCSST to characterise cartilage and to extract quantitative measures of subchondral bone integrity.

**Methods:**

Seven excised human knees (3 males and 4 females; 4 normal and 3 with osteoarthritis; age 80.6 ± 14 years, mean ± standard deviation) were scanned using a clinical PCSCT prototype scanner. Tomographic image reconstruction was performed after Compton/photoelectric decomposition. Virtual monoenergetic images were generated from 40 keV to 110 keV every 10 keV (cubic voxel size 250 × 250 × 250 μm^3^). After selecting an optimal virtual monoenergetic image, we analysed the grey level histograms of different tissues and extracted quantitative measurements on bone cysts.

**Results:**

The optimal monoenergetic images were obtained for 60 keV and 70 keV. Visual inspection revealed that these images provide sufficient spatial resolution and soft-tissue contrast to characterise surfaces, disruption, calcification of cartilage, bone osteophytes, and bone cysts. Analysis of attenuation *versus* energy revealed different energy fingerprint according to tissues. The volumes and numbers of bone cyst were quantified.

**Conclusions:**

Virtual monoenergetic images may provide direct visualisation of both cartilage and bone details. Thus, unenhanced PCSCT appears to be a new modality for characterising the knee joint with the potential to increase the diagnostic capability of computed tomography for joint diseases and osteoarthritis.

## Key points

- Photon-counting spectral compute tomography (PCSCT) is a new tool to explore joints with high spatial resolution.
- Virtual monoenergetic images at 60 keV and 70 keV provided sufficient soft-tissue contrast to characterise surfaces, disruption, calcification of cartilage, bone osteophytes, and bone cysts.
- On the PCSCT virtual monoenergetic images at 60 keV, volume and density of bone cysts can be quantified semiautomatically.

## Background

Osteoarthritis (OA) is a chronic inflammatory joint disorder characterised by cartilage loss, abnormal subchondral bone, osteophyte formation, degeneration of ligaments, meniscus, and hypertrophy of the capsule [1]. The damage to the cartilage is typically characterised by fissures in the superficial layers, which gradually extend to deeper zones, and finally full cartilage loss [2]. It is essential to detect early changes during the reversible phase of the disease. Progresses in OA management require the development of noninvasive diagnostic methods that can be used to quantify changes in the cartilage and subchondral bone [3].

Imaging methods used for diagnosis of OA are usually conventional radiography and magnetic resonance imaging, the latter being classically used in clinical routine to visualise joint effusion, cartilage, ligaments, tendons, meniscus, osteophytes, and bone marrow oedema.

Computed tomography (CT) is not ideally suited to the observation of soft tissue like cartilage, however when combined with intra-articular application of a contrast agent, it can be used to investigate the cartilage surface. Moreover, CT has been proposed for chondrocalcinosis diagnosis [4], and for quantification of both volume and number of subchondral bone cysts [5].

Dual-energy CT, combining measurements from two energy spectra has been shown for its use in musculoskeletal imaging particularly in the detection of gout [6]. However, a recent publication showed that this technique does not bring more information than conventional CT for calcium pyrophosphate deposition [7], and to the best of our knowledge, it is not used for cartilage analysis. Today, the new generations of photon-counting spectral CT (PCSCT) scanners include energy-discriminating photon-counting detectors (PCDs) that can simultaneously count photons and resolve their energy [8, 9] conversely to conventional CT scanners energy-integrating detectors. With such systems, it is possible to reconstruct different types of images like material decomposition images or virtual monoenergetic images. Another advantage of this new detector is the improved signal-to-noise ratio (SNR), due to the exclusion of electronic noise [10]. With such additional information, PCSCT is expected to surpass conventional CT and open new possibilities in medical diagnosis.

PCSCT is ideal for material decomposition imaging, as for K-edge imaging, which uses the discontinuity at diagnostic energies of the linear attenuation coefficient of high-Z element-based contrast agents, such as gadolinium, gold, and bismuth [11, 12]. Indeed, PCSCT combined with one or several contrast agents has been proposed for diverse applications. These included tracking and monitoring of the biodistribution of gold nanoparticles *in vivo* [13], determination of contrast agent concentrations in the liver [14], and evaluation of the risk of breast cancer [15]. PCSCT might also be used to quantify calcium content (*e.g.*, in bone, teeth, kidney stones, coronary plaques) and to discriminate between different calcium crystals [16, 17]. In addition to material decomposition and K-edge imaging, PCSCT offers the possibility to obtain virtual monoenergetic images, with the potential to increase the contrast especially for soft tissues [18]. This feature and the reduced detector pixel size provided by PCD makes PCSCT a new candidate for medical applications where resolution and soft tissue contrast are critical. Considering its applications to OA to date, PCSCT coupled to an iodine contrast agent has only been considered in one study, for measuring proteoglycan content in cartilage in knee specimens [19]. Thus, there are no current methods that have sufficiently high resolution and image quality to visualise the internal structures of the bone, the meniscus, and cartilage details at the same time [20].

Thus, the aim of this study was to investigate the feasibility of PCSCT for assessment of joint integrity without the need for a contrast agent. Our first aim is to evaluate whether virtual monoenergetic reconstructions produced sufficient spatial resolution and soft tissue contrast to visualise both cartilage and bone with enough details without contrast agent. Our second aim is to evaluate the quality of the images produced is sufficient to perform quantitative analysis.

## Methods

### Sample description

Seven knee specimens from 3 males and 4 females, aged 80.6 ± 14 years (mean ± standard deviation) were obtained from the Institut d’Anatomie Paris (France). The collection of these human tissue specimens was conducted according to the relevant protocols established by the Human Ethics Committee from the Institute of Medical Research. No additional information was available regarding cause of death, previous illnesses, or any medical treatments of these subjects except for an absence of hepatitis and human immunodeficiency virus. The protocol was approved by the French Ministry of Higher Education and Research (CODECOH number DC-2019-3422). After soft tissue removal, the knee specimens were stored at -20 °C.

### Data acquisition and image reconstruction

The knee specimens were imaged using a clinical PCSCT prototype system (Philips Healthcare, Amsterdam, Netherland) installed at CERMEP, Lyon. This is a modified clinical system that is equipped with a conventional X-ray tube that can be set to tube voltages from 80 to 120 kVp, and tube currents between 10 mA and 500 mA; it was set at 120 kVp and 100 mA in the present study. The tube filtration absorbs low-energy X-rays, so the final spectrum ranges from 30 keV to 120 keV. The system is based on PCDs of 2-mm-thick cadmium zinc telluride, with a pixel pitch of 270 × 270 μm^2^ at the isocenter, and coupled with application-specific integrated circuits (ChromAIX2, Philips Research Europe, Aachen, Germany) that operate in single-photon-counting mode with energy discrimination [21]. The acquisition time was less than 5 min. The PCDs allow up to five consecutive energy thresholds between 30 keV and 120 keV, which were set in the present study at 30, 51, 62, 72, and 81 keV. The acquisition field of view was 500 mm in-plane, with a *z*-coverage of 17.5 mm in the scanner isocenter. Axial scans were performed over 360° with 2,400 projections per rotation, on a grid of 64 × 1,848 pixels.

Fifty stacks of eight slices were acquired to cover the entire knee specimens (height, 10 cm). After data acquisition, the projections in the different energy bins were decomposed on a Compton/photoelectric basis using the maximum-likelihood method [11]*.* Then, the decomposed Compton/photoelectric sinograms (Radon transform) were reconstructed using filtered back-projection. The whole reconstructed images were made of 640 × 640 × 400 voxels, with a voxel size of 250 × 250 × 250 μm^3^ with a reconstructed scan field of view about 160 × 160 × 100 mm^3^. Seven virtual monoenergetic images from 40 keV to 110 keV were then computed from the linear combination of the reconstructed Compton/photoelectric images and expressed in Hounsfield units (HU) units. In addition, the conventional HU image obtained by combining all of the bins together was computed.

For comparison, we used a standard HR-pQCT imaging protocol (Scanco Wangen-Brüttisellen, Switzerland) with voxel size of 82 μm for all specimens, and synchrotron radiation monochromatic CT at 55 KeV (European Synchrotron Radiation Facility, Grenoble beamline ID 17) with a voxel size at 45 μm for 5 specimens. The Kellgren-Lawrence classification [22] was performed on the HR-pQCT images.

### Image analysis

First, we investigated the multienergy feature of PCSCT. For this purpose, we analysed the monoenergetic images and compared them to the conventional HU images to select an optimal monoenergetic image for cartilage assessment. This required selection of the energy that led to the best characterisation of the cartilage in terms of noise and contrast-to-noise ratio (CNR). The noise was computed as the standard deviation (SD) for a manually selected circular region of interest with radius of 4 pixels in a homogeneous part within the cartilage. The CNR was computed as the difference for the grey levels along a line inside the cartilage and a line inside the joint space outside the meniscus divided by the SD previously computed. The monoenergetic approach allows to retrieve the information that links the X-ray attenuation to the type of material crossed and the energy. We analysed the attenuation variation of cartilage, of both trabecular and cortical bone and soft tissue with energy for all knees.

The second part of the image analysis consisted of the qualitative assessment of OA from the monoenergetic images at the selected energy. For this, the feasibility of visual assessment of cartilage defects, cartilage calcification, bone cysts, and bone osteophytes was investigated by an expert radiologist who had been in practice for > 20 years (J.B.P.).

The third part concerned the extraction of quantitative parameters from the monoenergetic images at the selected energy. We analysed bone cysts observed in the monoenergetic images for the two severe OA knee specimens. To this aim, we performed the segmentation of the subchondral bone of the femur and patella in the femoro-patellar compartments to a total depth of 10 mm from the subchondral surface. The bone cysts were segmented using the commercially available Avizo 9.0 software (FEI Visualization Sciences Group, Burlington MA, Avizo v.9.0) with the semiautomatic region growing tool (Magic Wand). Here, a seed point was first defined by the operator, and all of the connected voxels with grey levels in a given tolerance range were selected. Any object with a volume < 20 voxels (0.31 mm^3^) was considered as noise, and was removed from the final calculations. The bone cysts in the medial and lateral compartments were analysed separately. After segmentation, the following parameters were measured: number of cysts/mm^3^, total cyst volume (mm^3^), and maximum cyst volume (mm^3^). Our quantitative analysis was exploratory, so not supported by statistical analysis.

## Results

### Multienergy imaging

**Fig. 1:**
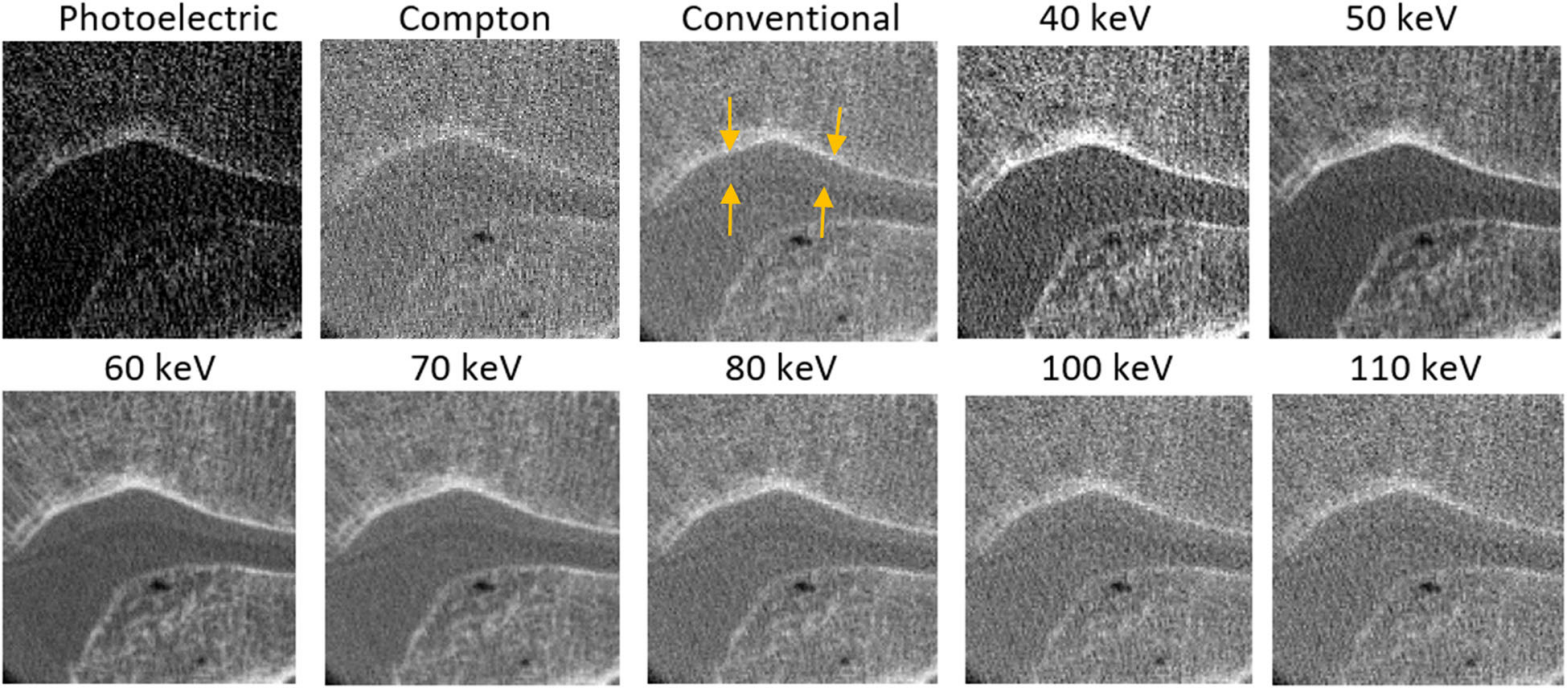
Details of the reconstructed images for the decomposed materials (photoelectric and Compton effects), conventional image (merging all energy bins), and virtual monoenergetic images (as indicated) for one normal specimen. Orange arrows, upper and lower surfaces of cartilage

Details from one normal specimen (sample 3) are presented here for the reconstructed images from the decomposed photoelectric and Compton images, the conventional HU image (from merging of all of the energy bins), and the virtual monoenergetic images, from 40 keV to 110 keV (Fig. 1). We note that the cartilage is visible in the Compton and conventional HU images, and in the virtual monoenergetic images above 50 keV. High-energy monoenergetic images above 80 KeV appeared to provide not only improved contrast, but also higher noise. Among all of these monoenergetic images, those for 60 keV and 70 keV appeared less noisy and with greater image quality than the Compton and conventional HU images.

**Fig. 2.**
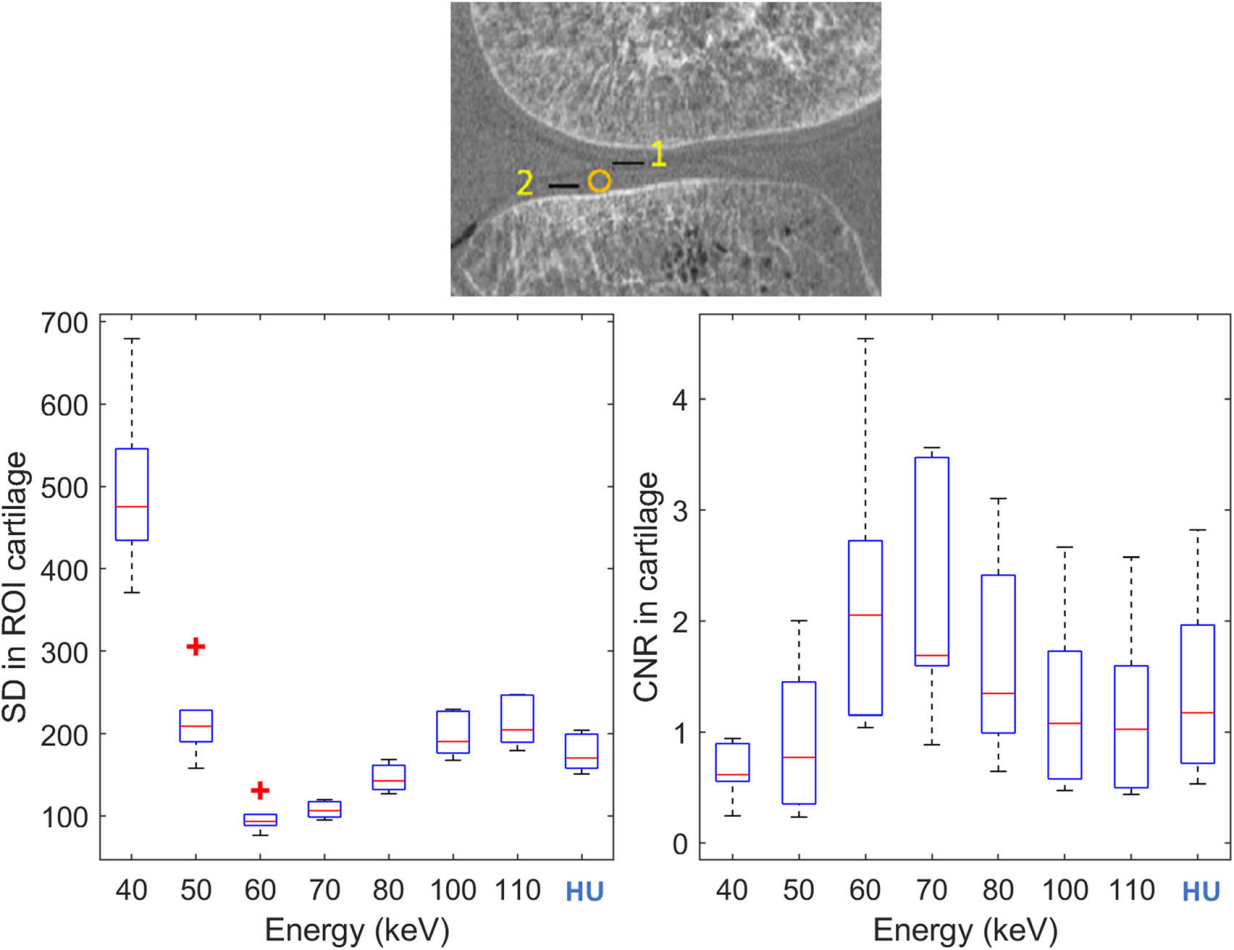
Noise standard deviation (SD) (left) and contrast-to-noise ratio (CNR) (right) for the cartilage for all of the monoenergetic images (40−110 keV) and the conventional image (HU). Plots display median and confidence intervals across the seven samples, with red crosses to indicate outliers. Top: Region-of-interest masks used for the analysis. Noise is computed as the SD within the circular region in the cartilage. CNR is computed as the contrast given by black lines *1* and *2* divided by the SD

The noise was computed as the SD inside the cartilage, and the CNR as the differences for the grey levels between the cartilage and the surrounding soft tissue for all of the monoenergetic images and the conventional images (Fig. 2). Among the monoenergetic images, 60 keV showed the lowest noise levels and the highest CNR, where the noise was a little lower than that at 70 keV. Adopting the conventional HU image as reference, the 60 keV monoenergetic image led to a 45% reduction in noise and 75% increase in CNR, which is relevant for good visualisation of cartilage.

**Fig. 3:**
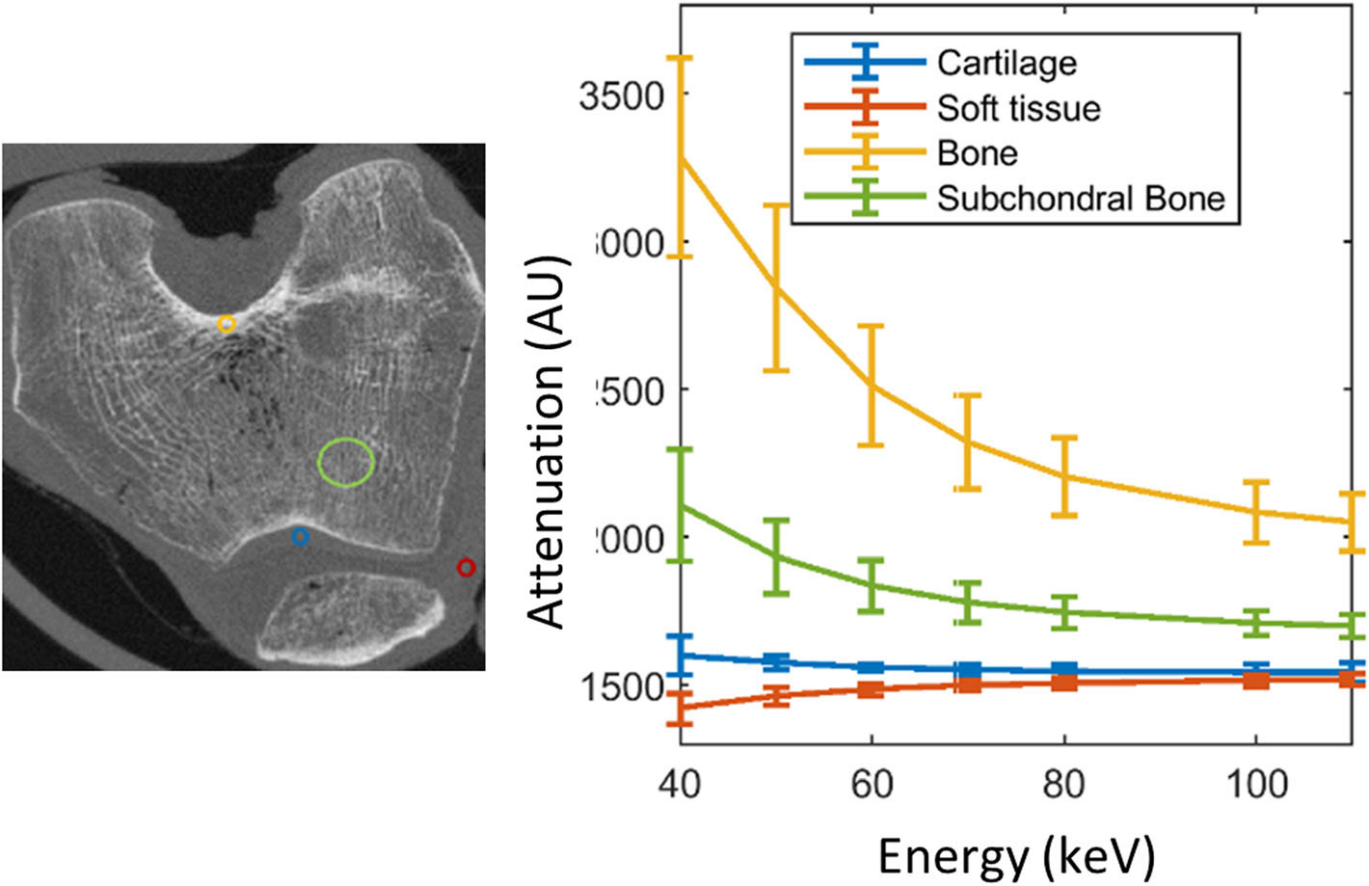
Right: Energy fingerprints of the different joint tissues computed from the virtual monoenergetic images. Attenuation *versus* energy for the regions of interest shown in the left panel: patella cartilage (blue), soft tissue different from cartilage (red), cortical bone (yellow), and subchondral bone (green). Plot shows mean and standard deviation across subjects for the selected regions of interest

Cartilage, soft tissue surrounding bone, and the different bone segments showed large variations in attenuation *versus* energy (Fig. 3). At 60 keV, the attenuation values ranged from 1,551 to 1,581 HU for cartilage and from 1,447 to 1,503 HU for soft tissue.

### Qualitative assessments of monoenergetic images

**Fig. 4:**
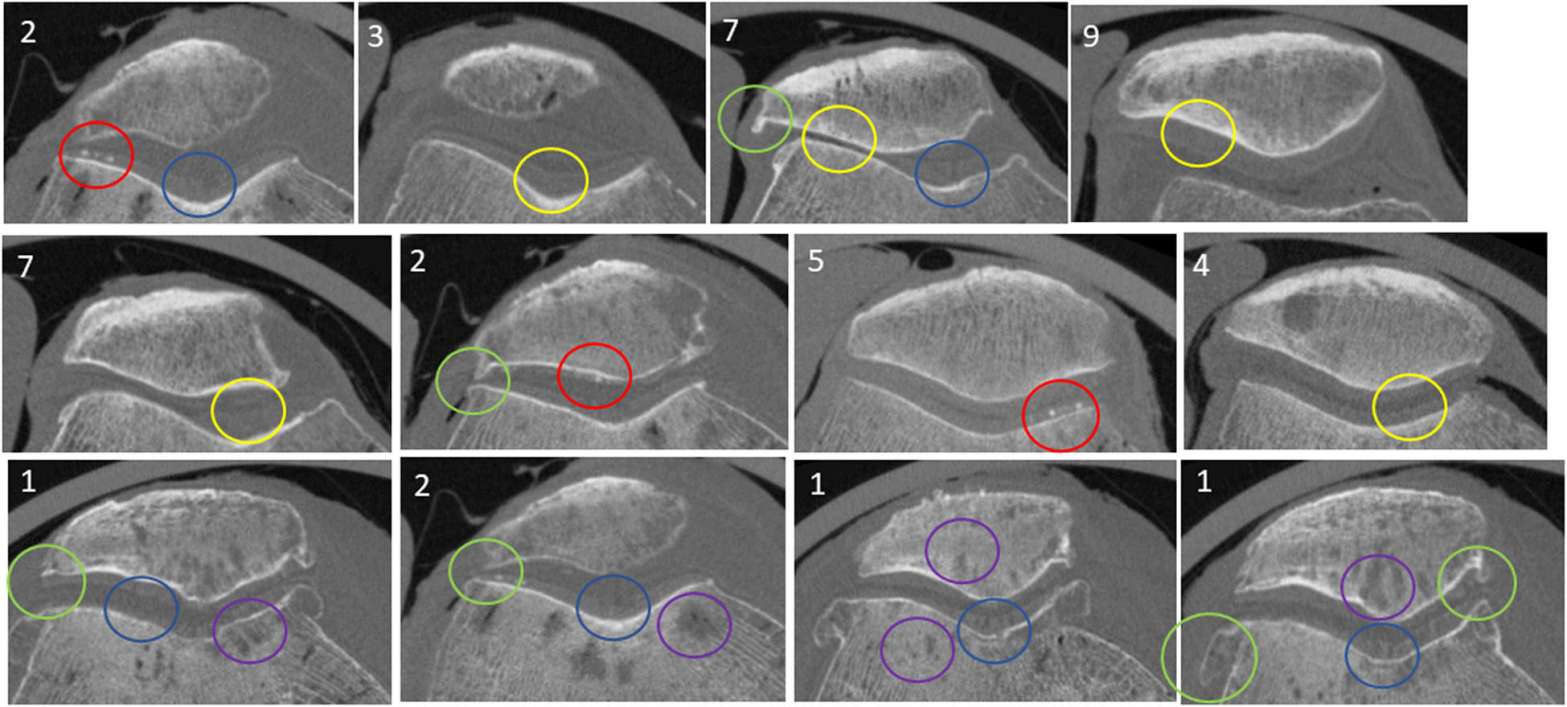
Transversal views of the selected 60 keV monoenergetic images for six specimens, the specimens 1 and 2 are severe osteoarthritis and sample 7 moderate osteoarthritis with numbers corresponding to samples. Red circle, cartilage calcifications; yellow circle, cartilage with different thicknesses; blue circle, cartilage defects; purple circle, bone cysts of various size; green circle: osteophytes of various size

The results of application of the Kellgren-Lawrence classification on the HR-pQCT images are described in Table 1. Cartilage defects qualified by a 75% local cartilage height loss, cartilage calcification, bone cysts are visible on the 60 keV monoenergetic images (Fig. 4). Bone osteophytes for subjects across the different levels of OA are pointed out (Fig. 4). Table 1 gives the clinical descriptions of all studied knee specimens relative to the cartilage aspects, osteophytes and subchondral bone cysts.

**Table 1:** Clinical descriptions of the knee specimens in terms of cartilage, osteophytes, and bone cyts

Sample	Sex	Age	KL	Lateral		Medial	
Cartilage				Femur	Patella	Femur	Patella
1	F	89	4	Thin, defects	Thin, defects calcifications+	Thin, defects	Thin, defects
2	M	89	3	Thin, irregular calcifications+	Thin, irregular calcifications+	Thin, defects calcifications+	Thin, irregular calcifications+
3	M	59	1	Normal, smooth	Normal, smooth	Normal, defects	Normal, smooth
4	F	90	0	Normal, smooth	Thin, defects	Normal, smooth	Normal, irregular calcifications+
5	F	81	0	Normal, smooth	Normal, smooth calcifications+	Normal, smooth calcifications+	Normal, smooth Calcifications+
6	F	94	1	Normal, irregular	Normal, irregular	Normal, smooth	Normal, smooth
7	M	63	2	Thin, defects	Thin, defects	Normal, defects	Thin, defects
**Subchondral bone cysts**							
1				Large size, numerous	Large size, numerous	Large size numerous	Large size, numerous
2				Middle size few	Small size numerous	Middle size few	–
3				–	–	–	–
4				Middle size numerous	Large size numerous	–	Middle size few
5				–	–	–	–
6				–	–	–	–
7				Small size few	Small size few	–	–
**Osteophytes**							
1				Large size	Small size	Large size	Large size
2				–	Small size	Small size	–
3				–	–	–	–
4				–	–	–	–
5				–	–	–	–
6				–	–	–	–
7				Small size	Small size	Small size	Small size

**Fig. 5:**
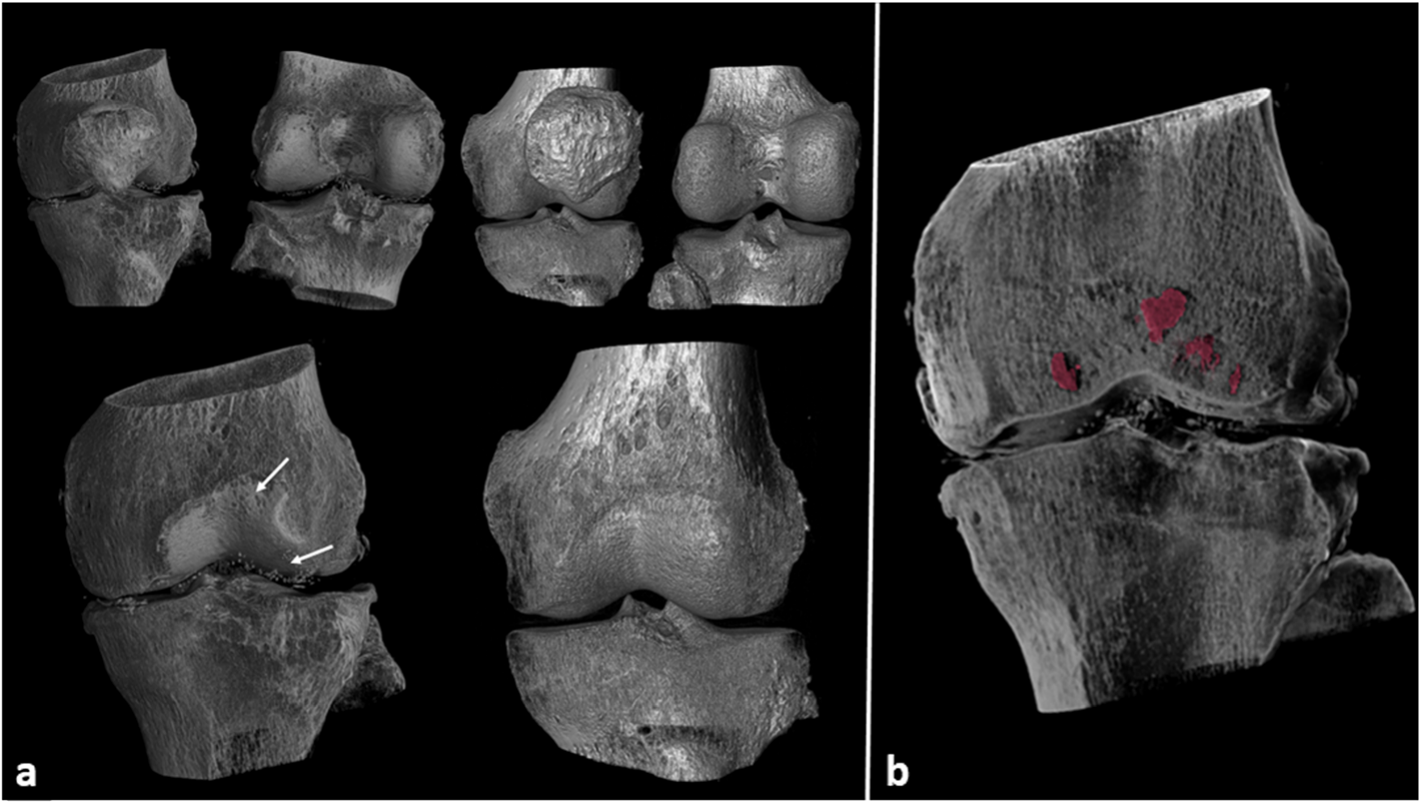
Three-dimensional displays of the 60 keV monoenergetic for two selected samples. **a,** left, OA (sample 2); **a**, right normal (sample 3). White arrows show cartilage defects (top) and calcifications (bottom) on OA. **b** Illustration of segmented bone cysts (in red) with the semiautomatic region growing tool in OA sample (sample 2)

Figure 5 shows the three-dimensional displays of the 60 keV monoenergetic images of two selected samples: left, OA (sample 2); right, normal (sample 3). The top images include the femur, the tibia, and the patella. In the bottom images, the patella has been numerically removed to visualise the cartilage surface. Indeed, cartilage surface defects and calcifications can be seen for the OA specimen (left), with no defect apparent for the normal specimen (right). Subchondral bone cysts segmentation of the OA specimen in the femur (sample 2) is also displayed on Fig. 5.

**Fig. 6:**
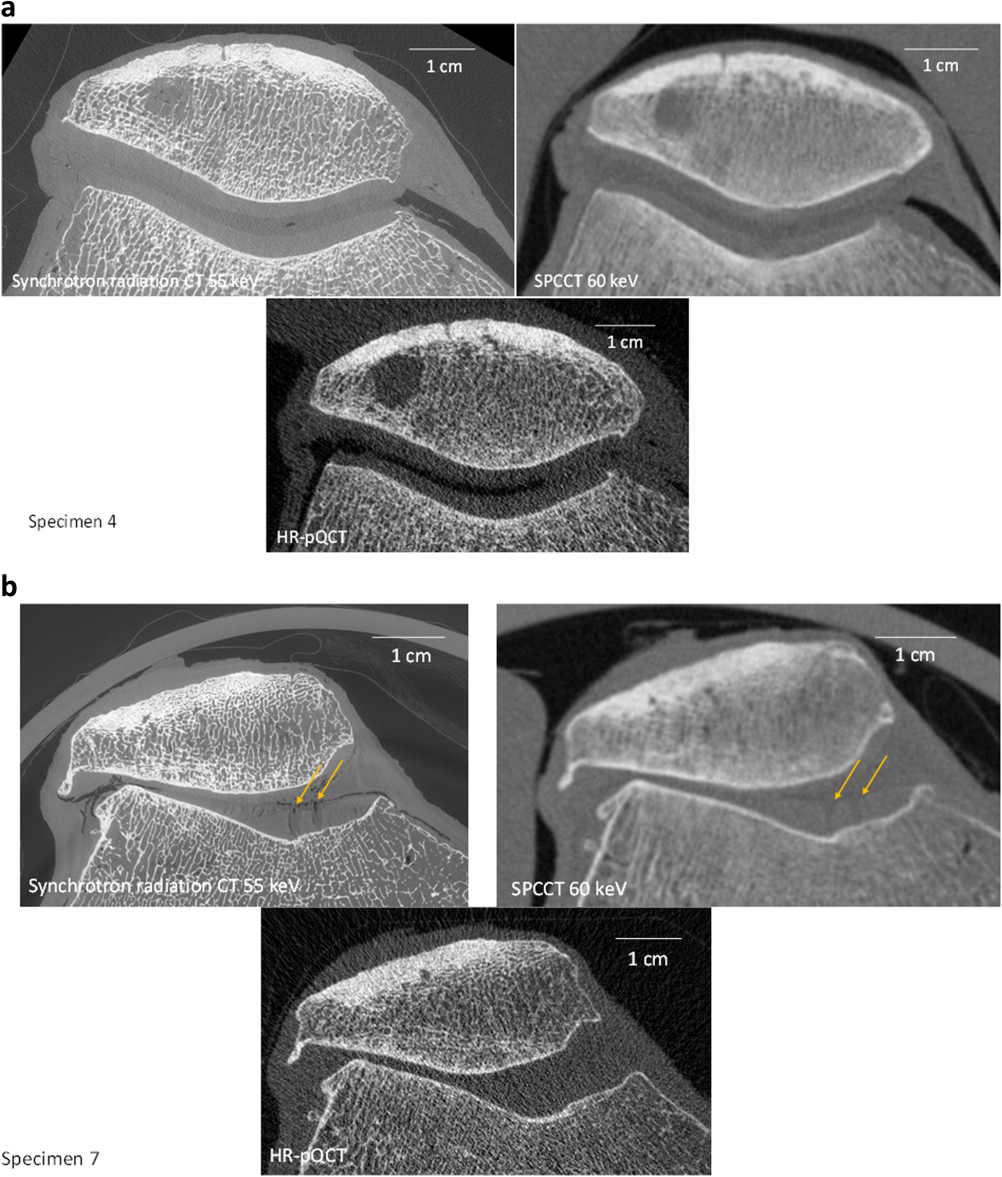
Synchrotron radiation images at 55 keV, PCSCT monoenergetic images at 60 keV and HR-pQCT images for 2 samples: a normal one (specimen 4); and an osteoarthritic one (specimen 7). The cartilage border with joint space is visible on the monoenergetic images based on synchrotron radiation, taken as references and the virtual PCSCT monoenergetic images. The yellow arrows correspond to cartilage defects

PCSCT allowed the visualisation of the cartilage with a quality approaching synchrotron radiation CT especially for the border of cartilage with joint space (Fig. 6). On the contrary, cartilage was not visible on conventional CT such as HR-pQCT with energy integrating detectors, even with high spatial resolution.

### Quantitative analysis of selected monoenergetic images

Bone cysts were found in two OA specimens (sample 1 and sample 2), and were segmented for both the femur and patella in a region up to 10 mm from the cartilage, to measure the quantitative parameters. The number and total volume of the bone cysts, and their maximal volume for each knee are given in Table 2. A three-dimensional display of the segmented bone cysts is provided in Fig. 5 (red) in sample 2.

**Table 2:** Quantitative analysis of the bone cysts for two of the specimens

Specimen	Site	Compartment	Cysts		
			Number	Volume (mm^**3**^)	
				Total	Maximum
Sample 1	Femoral	Medial	8	185.8	110.6
		Lateral	44	355	106.1
		**Total**	**52**	**540.8**	**110.6**
	Patellar	Medial	10	105.1	31
		Lateral	12	371	204.9
		**Total**	**22**	**476.1**	**204.9**
Sample 2	Femoral	Medial	3	241.8	109.5
		Lateral	4	223.2	125.5
		**Total**	**7**	**465**	**125.5**
	Patellar	Medial	4	13.2	5.5
		Lateral	11	37.9	17.7
		**Total**	**15**	**51.1**	**17.7**

## Discussion

Virtual monoenergetic images can provide direct visualisation of the details of the cartilage and bone on the same image. To the best of our knowledge, this is the first study to investigate PCSCT for OA without the need for any contrast agent. We have shown that the 60 keV and 70 keV monoenergetic images are optimal in terms of noise and CNR, respectively, and that these provide superior image quality compared to the HU images.

In a previous PCSCT study performed *in vivo* for the abdomen [18], 72 keV monoenergetic images showed improved contrast and SNR compared to conventional images with similar attenuation patterns. In the present study, the monoenergetic images in the range from 60 keV to 80 keV had lower noise than the conventional images, with the 60 keV and 70 keV monoenergetic images showing similar noise levels, although the lowest noise overall was for 60 keV. These data confirm the improvements in the SNR demonstrated in a previous simulated study that used a cadmium zinc telluride PCD in comparison to conventional detectors [21]. Indeed, the intrinsic qualities of PCDs can explain SNR and CNR increases: first, the detector counts the number of pulses greater than the preset threshold, which consequently eliminates the electronic noise; secondly, high energy photons contribute more than low energy photons in conventional detectors, which consequently decreases the CNR. In contrast, for PCDs, the equally weighted energy photons have positive effects on the contrast [9]. We hypothesise that the higher resolution and higher CNR in soft tissue for PCSCT with respect to standard CT will lead to superior diagnosis potential for applications for which resolution and contrast are critical. In addition, another major advantage of virtual monoenergetic images is that they provide quantitative attenuation measurements and reduce beam hardening artifacts [23]. Finally, one of the major advantages of PCD detectors is the patient’s radiation dose reduction. Indeed, based on an American College of Radiology accredited phantom, it was demonstrated that image quality can be maintained with a reduction in the dose of 40 to 60% [24].

Different joint tissues showed specific energy fingerprints. In particular, no overlap was seen between the cartilage and the soft tissue surrounding the cartilage. The analysis of the histograms showed that subjects with cartilage defects are shifted in the lower part of the histograms and on the contrary the presence of calcifications shifted the histogram in the high part.

Indeed, it is possible to discriminate calcifications in cartilage and the meniscus. CT has been proposed to evaluate calcium deposition, which is especially useful in the early stages of OA [24]. Using a preclinical scanner with high-resolution (*i.e.*, 0.1 mm^3^ voxels) and low-energy monoenergetic images (*i.e.*, 20−80 keV), it is possible to differentiate different calcium deposits in peripheral joints, such as calcium pyrophosphate and calcium apatite [16, 17] In the present study, PCSCT allowed the characterisation of bone cysts, which are related to mechanical stress in OA knees, and consequently this will be interesting to study further for better understanding of the OA process [25] and it will also help to better phenotype such OA patients [26] and cysts formation following cartilage repair [27]. Indeed, bone cysts quantification were rarely performed from standard CT [26] and one time from high-resolution peripheral CT [28].

The present study is subject to some limitations. The reconstructed images depend on the material decomposition and the postprocessing methods used. Material decomposition was carried out on a photoelectric and Compton basis. Other specific material basis, such as those based on bone and soft tissue, or even more general basis, are possible [29]. The investigation of other potentially improved basis might lead to further information with regard to the analysis of cartilage and calcifications. The material decomposition method used was based on conventional maximum likelihood, without regularisation in a pixel-by-pixel manner [11]. More advances inverse methods including regularisation [30–33] or based on deep learning approaches [34–36] might lead to improvements here. The image post-processing here was specifically designed for knee images; it might be further improved [37]. Our quantitative analysis is exploratory at this level, to show the potential of PCSCT for OA.

However, more refined methods in combination with a larger number of samples might lead to new biomarkers that translate into more accurate and meaningful OA scoring, and consequently to improved diagnosis. Further experiments carried out *in vivo* and with more subjects are needed to validate these findings.

In conclusion, virtual monoenergetic can provide direct visualisation of both cartilage and bone integrity, which are essential for the characterisation of osteoarticular diseases that affect the whole joint, with this demonstration thus focused on OA. In addition, these data suggest that quantitative measurements of bone cysts in terms of numbers and size might lead to new biomarkers for better phenotyping of OA. The prototype is operational for clinical research, with further studies needed to validate these data on living patients.

## Data Availability

The datasets used and/or analysed during the current study are available from the corresponding author on reasonable request.

## Abbreviations

CNR: Contrast-to-noise ratio
HU: Hounsfield unit
OA: Osteoarthritis
PCD: Photon-counting detectors
PCSCT: Photon-counting spectral computed tomography
SD: Standard deviation
SNR: Signal-to-noise ratio
